# Supplementary material for: Retrospective Analysis Revealed an April Occurrence of Monkeypox in the Czech Republic
Source: Viruses. 2022 Aug 15;14(8):1773. doi: 10.3390/v14081773 (PMC9412638; doi:10.3390/v14081773)
Supplement: Supplementary file 1 [file viruses-14-01773-s001.zip › Supplementary_information.pdf]

viruses

MDPI

Supplementary information

Retrospective analysis revealed an April occurrence of Monkeypox in the Czech Republic

Clinical and epidemiological report

The patient was a 38 years old male previously diagnosed with stage B1 human immunodeficiency virus (HIV) infection. Furthermore, other sexually transmitted diseases (STD) were also present in his history, including chlamydia, gonococcal infection and hepatitis C virus infection.

On 28th of April the patient was examined and hospitalized at the Department of Infectious Diseases of Military University Hospital, Prague. During the hospitalization the patient manifested with fever and papular rash with umbilications on the skin in the intergluteal area. A wide range of diseases, particularly STD, has been considered. Because of suspicion for herpes simplex virus type 2, although the clinical picture was not a typical herpetic rash, the patient was firstly treated with valaciclovir. Because of persisting fever and anal discomfort, the patient was then treated empirically with ceftriaxone and azithromycin due to suspicion of infection by *Neisseria gonorrhoeae* or *Chlamydia trachomatis*. Later, he was also treated locally with metronidazole suppositories. Meanwhile, the rectum of the patient was endoscopically examined and no pathological findings were observed. In the end, all the symptoms passed and the patient was dismissed.

Epidemiologically, the first symptoms of the disease were manifested on 24th of April 2022. Moreover, the patient claimed to have had unprotected sexual contact with a Caucasian male of unknown nationality on 16th of April, Lisbon, Portugal. Since he noticed that his sexual contact already manifested some signs of a disease (probably the Monkeypox), he did not allow any intimate contact except touching each other.

Transmission Electron Microscopy

The staining of the sample for Transmission Electron Microscopy (TEM) was basically performed according to Hayat [1]. Briefly, the formvar-coated carbon-reinforced copper grids (300 mesh) were hydrophilized by 1% Alcian blue in 1% acetic acid. The grids were then washed by brief contact with deionized water before they were floated on the drops of the virus suspension. After 10 minutes, each grid was successively washed by touching four droplets of deionized water. The grids were immediately stained by brief contact with a drop of 2% solution of phosphotungstic acid. Subsequently, the samples were examined and pictured using the TEM Jeol JEM, 200 CX at 80 kV.

Real-Time Polymerase Chain Reaction

First of all, in order to confirm the presence of the virus we performed Real-Time Polymerase Chain Reaction (qPCR) using the originally isolated DNA from the swab sample. We utilized Monkeypox specific primers and probe design targeting the A29L gene developed by Scaramozzino et al. [2]. Since both the methods, TEM and qPCR, confirmed the presence of an Orthopoxvirus in general, we decided to continue with more advanced methods that allowed detailed characterization of the sample in the view of molecular genetics/epidemiology, i.e. sequence analysis.

Virus cultivation

Patient isolate has been cultivated on Vero cell line, according to standard laboratory procedures [3, 4]. Briefly, 50 μL of the original swab was administered on the semiconfluent Vero cells in DMEM + 2% BOFES media supplemented with 200 IU/mL Penicillin and 200 mg/mL Streptomycine. The progress of infection was monitored by both qPCR and microscopic inspections. The supernatant containing virus was collected after 5 days and the total DNA was isolated and quantified according to standard procedures. The infected cell line underwent a total of one passage.

Library preparation and sequencing

All sequencing runs were performed using the Oxford Nanopore Technologies (ONT) GridION platform, which meets most of the criteria to be designated as Third Generation Sequencing [5]. Sequencing libraries were prepared according to the manufacturer´s guidelines (nanoporetech.com) including quantification of DNA on the Qubit fluorometer device (Invitrogen). We used the R9.4.1 sequencing chemistry (flow-cell) in combination with several ONT library preparation and barcoding kits (Ligation sequencing gDNA - native barcoding: SQK-NBD112.24).

Sequence data analysis

Sequencing data were basecalled, i.e. transmission from physical changes in electric current signal measured by the ONT sequencing device to biologically relevant bases, using Guppy v5.1.13 and the "high-accuracy" model (e.g. [6]). Resulting reads were mapped on Monkeypox virus reference (NCBI accession number ON568298.1) using minimap2 v2.17 [7]. Mapped reads were extracted using SAMtools v0.1.20 [8]. Such reads were then trimmed using Porechop v0.2.4 [9] with the option --discard-middle to get rid of reads with internal adapters, i.e. possible chimeras. The Monkeypox virus genome was assembled using Flye v2.9 [10] and polished by Medaka pipeline v1.5.0 [11]. The resulting sequence was deposited to GenBank under the accession number ON983168.1.

We have deliberately selected representative sequences across Europe to set our sample into the phylogenetic context of the current outbreak (see **Supplementary Table 1**). Multiple Sequence Alignment was conducted in the software MAFFT v7.505 [12]. Maximum Likelihood gene trees were reconstructed by IQ-TREE v2.2.0 [13] using the extended model selection with free rate of heterogeneity in combination with 1000 ultrafast bootstrap replicates [14-16]. The resulting tree was visualized using FigTree v1.4.4 [17]. Furthermore, the sequence was included into the Nextstrain on-line build (https://nextstrain.org/monkeypox/hmpxv1).

GenBank accession number	Country	Year
ON983168.1	Czech Rep.	2022
ON563414.3	USA	2022
ON585038.1	Portugal	2022
ON602722.2	France	2022
ON614676.1	Italy	2022
ON615424.1	Netherlands	2022
ON619835.2	UK	2022
ON622712.1	Belgium	2022
ON622718.1	Spain	2022
ON622720.1	Switzerland	2022
ON631963.1	Australia	2022
ON649879.1	Israel	2022
ON782021.1	Finland	2022
ON676708.1	USA	2021
MN648051.1	Israel	2018
MK783031.1	Nigeria	2017
MT903347.1	USA	2003

Supplementary Table S1: Samples included in the phylogenetic analysis, for each sample we provide GenBank accession number, country of origin and year of sample acquisition.

Supplementary References

1. Hayat MA, editor. Principles and techniques of electron microscopy: biological applications. 4th ed. Cambridge, UK; New York: Cambridge University Press; 2000. 543 p.

2. Scaramozzino N, Ferrier-Rembert A, Favier A laure, Rothlisberger C, Richard S, Crance JM, et al. Real-Time PCR to Identify Variola Virus or Other Human Pathogenic Orthopox Viruses. Clin Chem. 2007 Apr 1;53(4):606–13. doi: 10.1373/clinchem.2006.068635.

3. Wadell G. Cultivation of viruses. In: Textbook of Medical Virology. Elsevier; 1983. p. 38–44. doi:10.1016/B978-0-407-00253-1.50010-4.

4. Erez N, Achdout H, Milrot E, Schwartz Y, Wiener-Well Y, Paran N, et al. Diagnosis of Imported Monkeypox, Israel, 2018. Emerg Infect Dis. 2019 May;25(5):980–3. doi: 10.3201/eid2505.190076.

5. Schadt EE, Turner S, Kasarskis A. A window into third-generation sequencing. Hum Mol Genet. 2010 Oct 15;19(R2):R227–40. doi: 10.1093/hmg/ddq416.

6. Wick RR, Judd LM, Holt KE. Performance of neural network basecalling tools for Oxford Nanopore sequencing. Genome Biol. 2019 Dec;20(1):129. doi: 10.1186/s13059-019-1727-y.

7. Li H. Minimap2: pairwise alignment for nucleotide sequences. Birol I, editor. Bioinformatics. 2018 Sep 15;34(18):3094–100. doi: 10.1093/bioinformatics/bty191.

8. Li H, Handsaker B, Wysoker A, Fennell T, Ruan J, Homer N, et al. The Sequence Alignment/Map format and SAMtools. Bioinformatics. 2009 Aug 15;25(16):2078–9. doi: 10.1093/bioinformatics/btp352.

9. Wick RR, Judd LM, Gorrie CL, Holt KE. Completing bacterial genome assemblies with multiplex MinION sequencing. Microb. Genomics. 2017 Oct 1;3(10). doi: 10.1099/mgen.0.000132.

10. Kolmogorov M, Yuan J, Lin Y, Pevzner PA. Assembly of long, error-prone reads using repeat graphs. Nat Biotechnol. 2019 May;37(5):540–6. doi: 10.1038/s41587-019-0072-8.

11. Medaka: Sequence correction provided by ONT Research. Available from: https://github.com/nanoporetech/medaka (accessed on July 10, 2022)

12. Katoh K, Standley DM. MAFFT Multiple Sequence Alignment Software Version 7: Improvements in Performance and Usability. Mol Biol Evol. 2013 Apr 1;30(4):772–80. doi: 10.1093/molbev/mst010.

13. Nguyen LT, Schmidt HA, von Haeseler A, Minh BQ. IQ-TREE: A Fast and Effective Stochastic Algorithm for Estimating Maximum-Likelihood Phylogenies. Mol Biol Evol. 2015 Jan;32(1):268–74. doi: 10.1093/molbev/msu300.

14. Kalyaanamoorthy S, Minh BQ, Wong TKF, von Haeseler A, Jermiin LS. ModelFinder: fast model selection for accurate phylogenetic estimates. Nat Methods. 2017 Jun;14(6):587–9. doi: 10.1038/nmeth.4285.

15. Minh BQ, Nguyen MAT, von Haeseler A. Ultrafast Approximation for Phylogenetic Bootstrap. Mol Biol Evol. 2013 May 1;30(5):1188–95. doi: 10.1093/molbev/mst024.

16. Wang HC, Minh BQ, Susko E, Roger AJ. Modeling Site Heterogeneity with Posterior Mean Site Frequency Profiles Accelerates Accurate Phylogenomic Estimation. Syst Biol. 2018 Mar 1;67(2):216–35. doi: 10.1093/sysbio/syx068.

17. FigTree. Available from: http://tree.bio.ed.ac.uk/software/figtree/ (accessed on July 10, 2022)
